# Validation of Segmented Brain Tumor from MRI Images Using 3D Printing

**DOI:** 10.31557/APJCP.2021.22.2.523

**Published:** 2021-02

**Authors:** Ujwal Ashok Nayak, Mamatha Balachandra, Manjunath K N, Rajendra Kurady

**Affiliations:** 1 *Department of Computer Science and Engineering, Manipal Institute of Technology, Manipal Academy of Higher Education, Manipal, 576104, India. *; 2 *Research and Development, RTWO Healthcare Private LLP, Mahalakshmipuram, Bengaluru, 560086, India. *

**Keywords:** Medical image analysis, image processing, image segmentation, 3D printing

## Abstract

**Background:**

Early diagnosis of a brain tumor is important for improving the treatment possibilities. Manually segmenting the tumor from the volumetric data is time-consuming, and the visualization of the tumor is rather challenging.

**Methods:**

This paper proposes a user-guided brain tumour segmentation from MRI (Magnetic Resonance Imaging) images developed using Medical Imaging Interaction Toolkit (MITK) and printing the segmented object using the 3D printer for tumour quantification. The proposed method includes segmenting the tumour interactively using connected threshold method, then printing the physical object from the segmented volume of interest. Then the distance between two voxels was measured using electronic callipers on the 3D volume in a specific direction. And next, the same distance was measured in the same direction on the 3D printed object.

**Results:**

The technique was tested with n=5 samples (20 readings) of brain MRI images from RIDER Neuro MRI dataset of National Cancer Institute. MITK provides various tools that enable image visualization, registration, and contouring. We were able to achieve the same measurements using both the approaches and this has been tested statistically with paired t-test method. Through this and the observer’s opinion, the accuracy of the segmentation was proved.

**Conclusion:**

When the difference in measurement of tumor volume through the electronic calipers and with 3D printed object equates to zero, proves that the segmentation technique is accurate. This helps to delineate the tumor more accurately during radio therapy.

## Introduction

**Figure 1:**
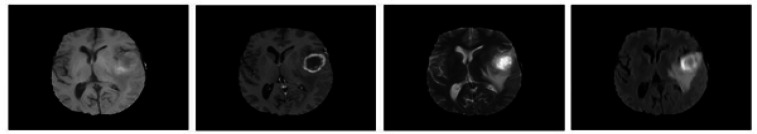
Four Different MRI Modalities are Showing a High-Grade Glioma, each Enhancing Different Sub-Regions of the Tumor. From left; T1, T1-Gd, T2, and FLAIR. Images are generated by using Brain Tumor Segmentation (BRATS) 2013 dataset [3, p. 319].

Early diagnosis of a brain tumor is important for improving the treatment possibilities. The anatomical imaging modalities like computed tomography (CT) and MRI (magnetic resonance imaging) and the functional imaging modalities like PET and SPECT are mainly used for tumor diagnosis. MRI creates various contrast levels of the tissue, providing various details of a structure. Some of the modalities of MRI for brain tumor diagnosis are T1-weighted MRI, T2-weighted MRI, with gadolinium contrast enhancement (T1-Gd) and fluid-attenuated inversion recovery (FLAIR) respectively (Figure 1a-d). Benign tumors show up non-uniform tissue intensities when compared with normal tissues, they appear darker or sometimes the same intensity on T1, and brighter on T2. Pressure areas where the brain tissue occluded by a tumor also appear brighter on T2 (Bauer et al., 2013). Generally, T1 images distinguish healthy tissues; edema region is separated using T2, tumor border is identified using T1-Gd and FLAIR helps in separating the edema region from cerebrospinal fluids. The contrast between the different types of modalities gives a unique signature to each tissue type. The challenges for brain tumor segmentation are mainly due to diversity in size, location, irregular shape, and their heterogeneous levels of tissue intensities of the tumor (Wang, 2018). Present state-of-the-art techniques involve manually segmenting the tumor through contouring tools. Manually segmenting the boundary of the tumor in all three MPR (Multi-planar reformation) planes is a laborious task. In addition to the segmentation, accuracy of validating the results is too less at present. 3D printing is an emerging technology to create physical objects from the software. The use of 3D printing technology has also been rapidly increasing in Medical imaging domain. Understanding the anatomy of the brain and other internal organs is easier when it is 3D printed.

The rationale behind the study is to find a solution wherein we can validate the segmentation results of the computer algorithms. At present, the segmentation results are validated only through statistical analysis by comparing the results with ground truth defined by the doctor. Proposed research is an alternative technique which involves Object->Scan->Segment->PrintObject and validate through Object and PrintedObject.

In this section, a brief overview of existing works in brain tumour segmentation is presented. Initially, image segmentation was done manually, where radiologists used information from the medical images along with their knowledge and expertise gained through training and experience (Heuristic approach)to segment the medical images (Isin et al., 2016). This was a time consuming and tedious task and also resulted in variable results based on the human performing the segmentation. Semi-automatic methods were introduced to reduce the drawbacks of manual segmentation (Udupa and Herman, 2000). This involved an algorithm which performed segmentation by defining a region of interest (ROI) and then adjustments were made as desired. Hamamci et al., (2012) proposed a semi-automatic method called tumour cut, which required the user had to draw the diameter of the tumour manually on the input image. A novel classification approach (Havaei et al., 2016) was proposed recently, which transformed a segmentation problem into a classification problem. Kwon et al., (2014) proposed a semi-automatic brain tumour segmentation technique that used a method which allowed multiple seeds points to grow each focal mass and combined them into a single density for classification into a tumour or edema. Zhao et al., (2013) introduced an approach to segmentation by labelling the volume slice-by-slice using Markov Random Field (MRF) to minimalize the energy on neighbouring slices. By manually labelling one slice, which is selected randomly, all other slices were iteratively labelled using MRF for energy minimization. Bahadure et al., (2016) used Watershed segmentation and Fuzzy C-means (FCM) clustering, which was an improvement of FCM based segmentation using the histogram. Semi-automatic methods are faster than manual methods but still provide variable results due to human intervention. Therefore, current research is focused on automating the segmentation procedure (Havaei et al., 2017; Xue et al., 2018; Kamnitsas et al., 2017; Pereora et al., 2016). Semi-automatic methods of segmentation require human expertise to properly segment the tumour from the Region of Interest (ROI). Therefore, the results depend on the strategy used by the user involved in the segmentation process as well as the algorithmic accuracy (Gordillo et al., 2013). There can be variations between the observer’s opinions in evaluating the segmentation results. To know the progress of the tumour growth patient is scanned over a period of time, and the tumour growth is studied. Toolkits that supported both manual, as well as semi-automatic segmentation techniques in the medical imaging domain, were introduced for easier and better image analysis, registration, and segmentation. Some of these toolkits are Insight Segmentation and Registration Toolkit (ITK) Visualization Toolkit (VTK), and MITK (DKFZ, Germany) (Yushkevich et al., 2018). MITK combines ITK, VTK, and Common Toolkit (CTK) with an application framework. MITK provides several tools for medical image segmentation like a livewire, region growing, or simply contouring. It also supports interpolation, cropping of surfaces, surface creation, histogram functions, and statistical analysis. Segmenting the tumour from a three-dimensional volume is a rather challenging and tedious task. These factors motivate this research to provide an efficient automated solution for segmentation of the tumour from the input MRI image and then reconstruct the segmented tumour through 3D printing to calculate the tumour mass.

There are many other good segmentation techniques like, K - Means clustering, Level set method, Fuzzy C thresholding, volume thresholding, gradient based edge detection methods and Principal curvature and confidence connected region growing technique. Even though they perform better in specific cases of radiology images and particular anatomies (they are good for CT images and hard structures like bones and uniform tissue intensities in the region of interest), the limitations is, the solution is not ubiquitous and they might not give good results in the case of brain MRI images as the anatomical structure details are fuzzy in nature and not the hard structures. We have excluded the discussion on such methods and have considered only the segmentation methods specific to brain MRI images.

## Materials and Methods

For the study, the brain MRI images were downloaded from the National Cancer Institute, USA (Daniel, 2015; Clark et. al., 2013). The dataset collection is RIDER Neuro MRI, which contains imaging data on 19 patients with recurrent glioblastoma who underwent repeat imaging sets. The images were acquired with 1.5T imaging magnet, and the slice thickness was 5mm. MRI scanning parameters are scanning sequence = {IR, EP}, Scan options = {PER, RG, PFF}, MR acquisition type ={3D, 2D}, Imaged nucleus = {1H, 31P}, Image type = { PHASE MAP, MPR, T1 MAP, T2 MAP}, Spatial resolution = {1.47 – 1.87 mm}, Flip angle = {90, 85, 95, 105, 110}. Few of the popular datasets for brain MRI are shown in Table 1. Challenges have been held annually to encourage better automated state-of-the-art methods for brain tumour segmentation [ISM]. Medical Imaging Interaction Toolkit (MITK) is an open-source software which can be used as a framework to develop sophisticated imaging applications in the medical domain. FDM (fused deposition modelling) technic and PLA (Polylactic acid) material were used for 3D printing.

**Figure 2:**
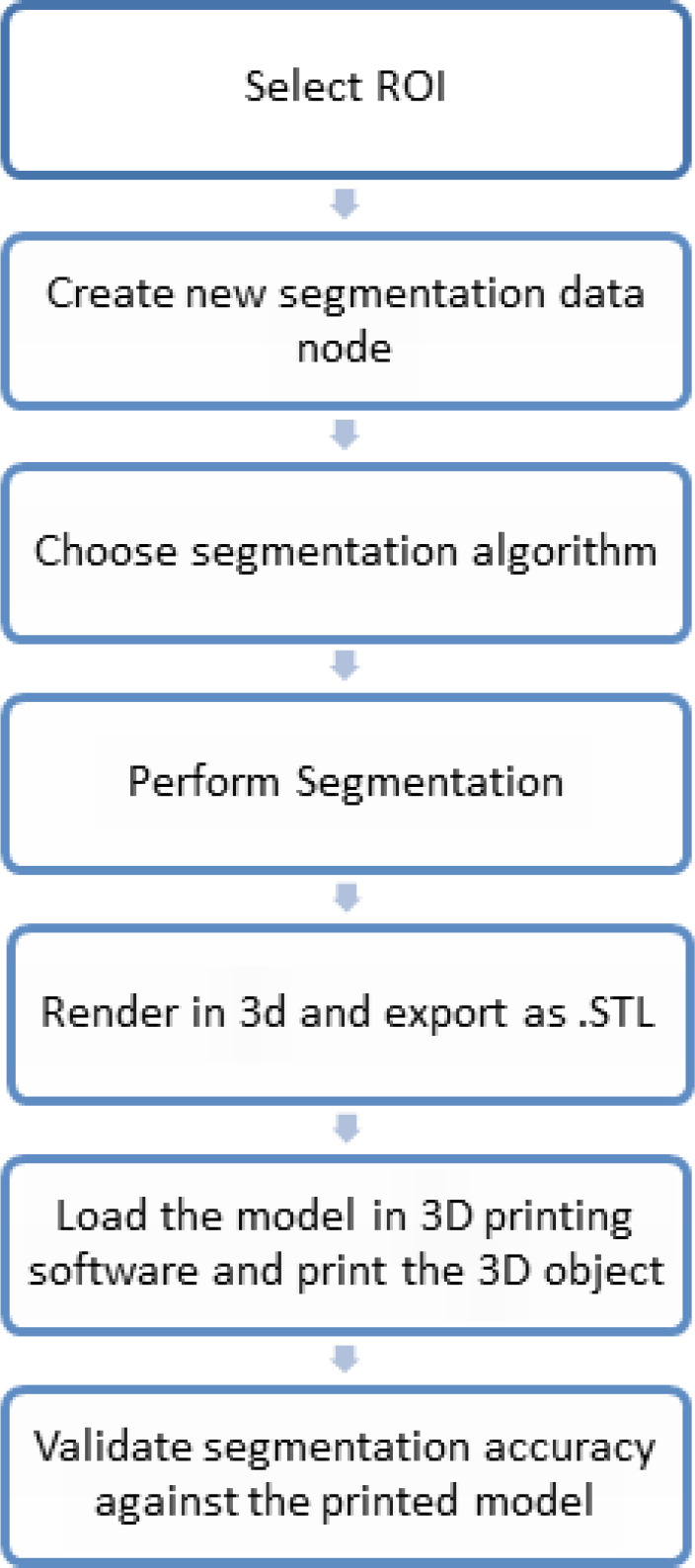
Workflow for Brain Tumor Segmentation

**Figure 3:**
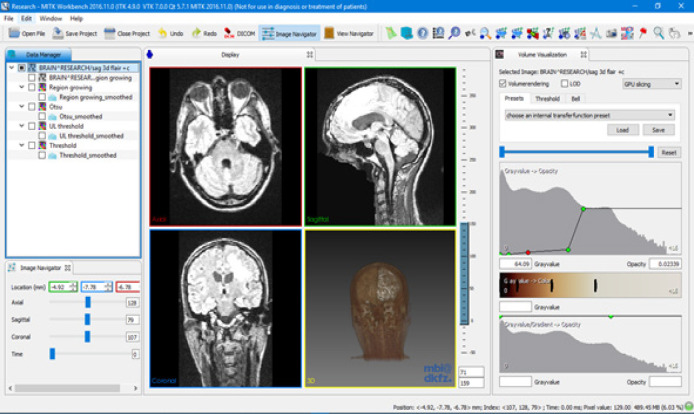
The User Interfaces in MITK Displaying the Orthogonal Slices of MRI in MPR Mode and the 3D View of the Volume in the Lower Right Quadrant

The workflow of the proposed technique is illustrated in Figure 2. The work was implemented using C++ with open-source frameworks ITK, VTK, and MITK. MITK supports DICOM, Neuroimaging Informatics Technology Initiative (NIFTI), and MetaImage formats. MITK provides various manual and semi-automatic tools for segmentation, visualization of 3D volume using iso-surface extraction technique and the multi-planar reformatted images in three axes of the patient coordinate system (slice-based visualization technique) (Figure 3).

Interaction in MITK is based on the state-machine concept. Events from different input devices are mapped to functions. A 3D crosshair 3D cross hair which represents the intersection of three orthogonal slices is defined, which is the intersection point of the three orthogonal slices. This feature is used to navigate the 3D volume on MPR segments. Segmentation is shown as an overlay which is usually a binary image overlapped on the input image. Semi-automatic segmentation starts with the user defining a seed point on the region of interest (ROI). Image cropper tool is used to select the ROI for accurate segmentation. ROI is selected by masking the image using a bounding box which the user can select explicitly.

Once the ROI mask is created, the segmentation algorithm can be applied onto the mask instead of the entire image data. Masking is done in 3D, so adjustments have to be made in all three dimensions to fit the bounding box. Some of the segmentation methods implemented are described below.

*A. Segmentation methods*

*Region growing*

**Figure 4:**
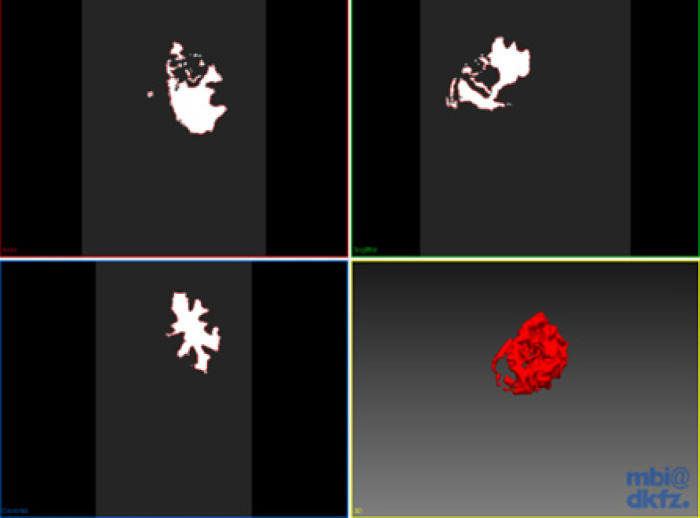
Segmented Brain Using Region Growing with Retained Voxel Intensities within the Segmented Area (Three MPR Planes and the Surface Rendered Image on the Right Side

This method has been widely used because of its simplicity. The algorithm uses neighbouring concept where a seed region (typically one or more pixels) is defined inside the object to be segmented. The pixel values of the neighbouring pixels are then evaluated to see if they are part of the same object. Region growing algorithms differ based on the strategy used to determine similar neighbouring pixels. The MPR views and the surface rendered image of region growing are shown in Figure 4.

*Connected Threshold*

**Figure 5:**
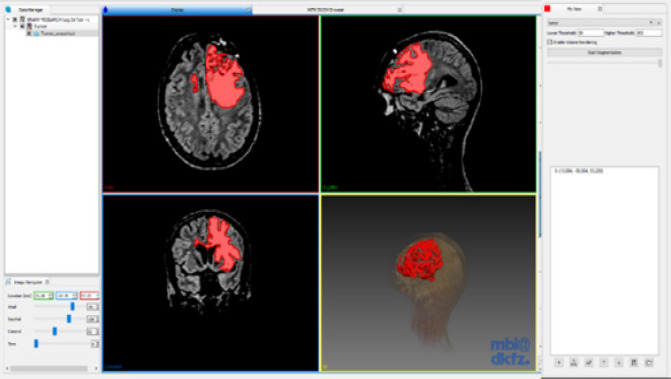
Segmentation of Tumor Using Connected Threshold Algorithm in Axial, Sagittal and Coronal Views along with the Surface Rendered Image

Thresholding operation identifies values of pixel-based on specifying one or more threshold values. Higher and lower threshold limits are to be provided. The region growing algorithm includes pixels whose intensity levels are within the given threshold. One such commonly used technique is called the connected threshold method. This technique uses a flood fill iterator wherein a seed point is selected, which is the starting point for the region to grow. An additional smoothing procedure is also applied to reduce the noise from the input image for a better region growing accuracy. The output of this will be a binary image where the pixel values of the background will be zero, and the segmented object will have a pixel value of one (Figure 5). One variant of the connected threshold is the neighbourhood connected method.

⋃ni=1Ri=R where Ri is connected

$P_{i}\cap R_{j}=\Phi \forall =1,2,\ldots ,n$

$P\left(R_{i}\right)=\mathrm{TRUE}\forall i=1,2,\ldots ,n$

$P\left(R_{i}\cup R_{j}\right)=\mathrm{FALSE}\forall j=1,2,\ldots ,n;i\neq j$

(1)

This technique uses a user-defined neighbourhood radius surrounding the pixel, where the pixel intensity of the neighbour should be within the threshold interval. Neighbourhood pixels are considered so that small structures are less likely accepted in the segmentation. Another variant is the confidence connected technique where the algorithm computes the mean and standard deviation of pixel values. A multiplier is used as input which calculates the range of pixels to be segmented. This is an iterative procedure which is repeated for every region by calculating mean and standard deviation and evaluate whether it falls into the range.

*Otsu’s algorithm*

Another way of segmentation is automatically finding the optimal threshold value by observing the distributed pixel values. Otsu’s segmentation (Wang et al., 2017) finds this threshold that classifies the image into multiple clusters so that intraclass variance is minimized. Using the histogram of the image, we get the spread of intensities of the pixel values which is used to classify them based on the number of clusters the user specifies.

$\sigma_{\omega }^{2}=\omega_{0}\left(t\right)\sigma_{0}^{2}\left(t\right)+\omega_{1}(t)\sigma_{1}^{2}(t)$

$\omega_{0}\left(t\right)=\sum_{i=0}^{t-1}p(i)$

$\omega_{1}\left(t\right)=\sum_{i=0}^{L-1}p(i)$

 (2)

**Figure 6:**
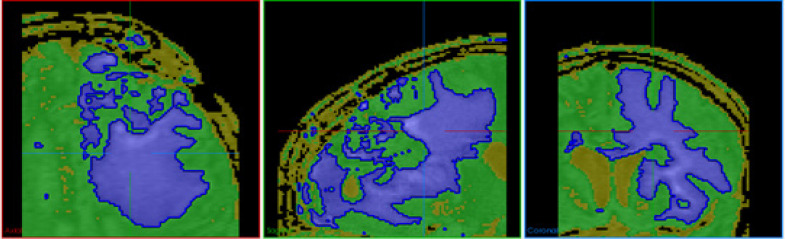
Classification of Pixels into Four Clusters (Blue Color) Using Otsu’s Algorithm in Axial, Sagittal, and Coronal Views Respectively. The crosshair shows the specific voxel location in 3D volume in all three MPR planes

**Figure 7:**
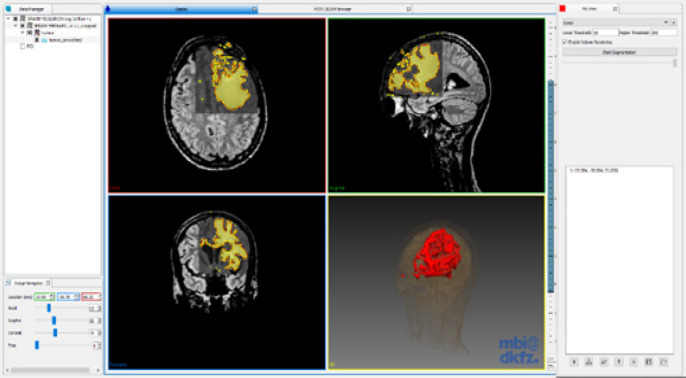
Segmentation Using Otsu’s Algorithm in Axial, Sagittal, and Coronal Views along with Surface Rendered Image. The segmented area is shown in yellow color on MPR images

Figure 6 and Figure 7 illustrates Otsu’s segmentation, which classifies the pixel into four clusters. Otsu thresholding is normally used for images having bimodal distribution wherein the intensity values can easily be separated into classes. Noisy images give rise to faulty segmentation in this case. So additional pre-processing has to be done to remove noise. We use a method where multiple threshold values are calculated for the given input histogram to maximize inter-class variance. ω_0_ and ω_1_ are the probabilities p(i) of the classes separated by threshold t and σ_0_^2^, and σ_1_^2^ are the variances of the classes. This is an iterative procedure and will repeat for all slices of the MRI. The output of segmentation is a binary image which is used to mask the original MRI to obtain the segmented image with retained intensity levels shown in Figure 4.

*B. 3D Printing*

The use of 3D printing technology has also been rapidly increasing in the medical imaging domain. Post segmentation, the 3D object was saved as STL (stereo lithography file format) object, which is the input for 3D printing. The object saves the boundary details similar to an XML format with the geometrical location of each boundary point. This STL object is used as input for the CURA software which prepares the model for 3D printing (Kumar et al., 2018). In the software, supports are added for surfaces that overhang. The Printer is initialized, materials and layer thickness is set, and additional settings are done as per user requirement. Here, the FDM technique and PLA as the material to print the model are used. It creates 3-dimensional objects by solidifying or joining liquid molecules. This was initially used for prototyping because of the material limitation. Factors like time taken for printing, material selection, cost of the printer are major concerns. FDM is used for models where a high level of detail is not required.

3D printing is done layer by layer by depositing binder material with inkjet printer heads. The models in STL format have to be checked for errors before printing. The types of errors that can occur are holes, noise shells, faces normal, manifold errors, self-intersections. STL generated through 3D scanning are more prone to these errors. Wall thickness is an important analysis check which measures the distance between surfaces of the model. A minimum of 1mm wall thickness is necessary for objects that would have an approximate size of 250 x 250 x 300 mm. Intricate detail analysis is finding details in the part which may not be printed accurately due to limitations of the type of printer or material. Support structures are necessary to print details which do not have base support (overhangs). Sometimes the part to be printed contains overhangs resulting in hard to remove supports. This issue also needs to be considered. The layer thickness is determined by the resolution of the printer in dpi or µm. Normally layer thickness is about 100 µm (250 dpi). High resolution will result in large file sizes without an increase in the quality of the print. Infill density is also a measure that has to be considered, which determines the strength of the object be printed. Objects that are created using FDM techniques are not completely solid because it would take very long to print. Only the outer shell of the model is printed. Infill density is the amount that is printed inside the model which is responsible for the weight and strength of the model. Higher infill density will result in a longer duration for printing.

**Table 1:** The Comparison of Different Metrics Calculated in the Empirical Testing (Only Ten Datasets are Shown)

Database name	URL
Brain Tumor Segmentation (BRATS)	https://www.smir.ch/BRATS/Start2013
Reference Image Database to Evaluate Therapy Response (RIDER)	https://wiki.cancerimagingarchive.net/display/Public/RIDER+NEURO+MRI
Mild Traumatic Brain Injury Outcome Prediction (mTop)	https://www.smir.ch/MTOP/Start2016/
Ischemic Stroke Lesion Segmentation (ISLES)	https://www.isles-challenge.org/
Multiple Sclerosis Segmentation (MSSEG)	https://www.nitrc.org/projects/msseg/
Neonatal Brain Segmentation (NeoBrainS12)	https://neobrains12.isi.uu.nl/

**Table 2:** The Measurement of Tumor and Anatomy (in cm) together under Both the Approaches

Sl.no	1	2	3	4	5	6	7	8	9	10
*Xi*	5.66	8.22	7.25	4.1	5.12	10.3	4	7.93	9.12	2.1
*Yi*	5.62	8.2	7.22	4.11	5.1	10.32	4.1	7.9	9.14	2.1
Sl. No.	11	12	13	14	15	16	17	18	19	20
*Xi*	4.11	8.33	9.46	8.22	4.28	11.5	7.3	9.2	7.1	6.3
*Yi*	4.1	8.34	9.44	8.2	4.3	11.4	7.4	9.22	7.11	6.2

**Figure 8:**
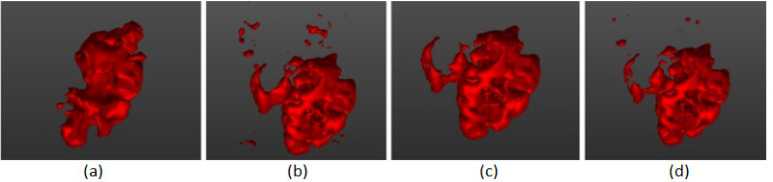
The Results of Different Segmentation Techniques Rendered with Surface Rendering Technique Using a Marching Cube Algorithm. (a) fast marching (b) Otsu (c) region growing and (d) connected threshold

**Figure 9:**
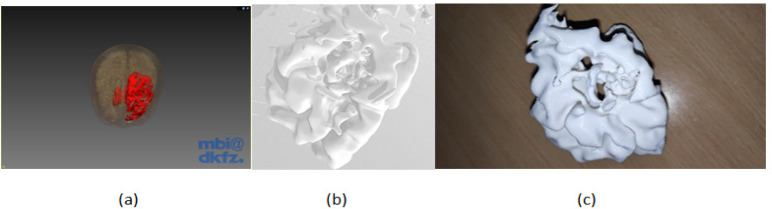
The 3D Model Viewed in the Same Direction. (a) Surface rendered image after segmentation using connected threshold technique, (b) the visualization of the 3D segmented object in STL format in windows application, and (c) the physical model after 3D printing

**Figure 10:**
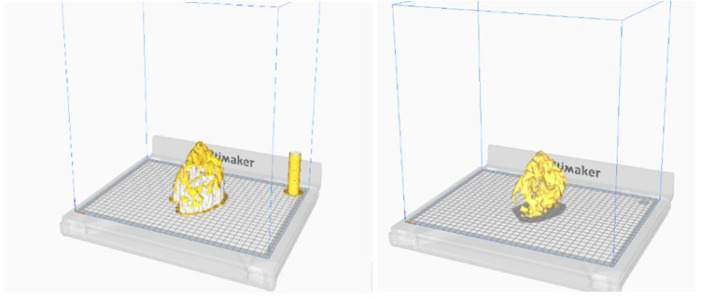
Brain Tumor STL Model without Supports (top) and after Adding Supports (Bottom) before Printing. This Simulation is Achieved in the Software before Printing the 3D Object Physically

## Results

The method was applied to MRI images of patients diagnosed with brain tumour obtained from RIDER dataset (Clark et al., 2013). Segmenting the tumour region from the MRI took less than five seconds (CPU implementation) in the connected threshold method and about twenty seconds in Otsu’s segmentation method, which is considerably faster than segmenting the tumour manually. Both algorithms could identify pixels of similar intensities and identify the region in which tumour mass was present. After performing various segmentation techniques, the best method was sifted (connected threshold) by visually inspecting the 3D surface rendered image (Figure 8, a-d). We looked into the closure ness and connectedness of the segmented boundary to make sure that there is no discontinuity in the boundary information.

Figure 10 shows the created STL model, which is used for 3D printing. The object in Figure 9 is of size 77.81 mm x 99.18 mm x 83.11 mm and contains 95565 faces and 17 shells. The model took eight hours for printing.

Once the 3D object is printed, it is compared with the original tumour model to check the accuracy of segmentation by randomly measuring the distance between the voxels on 2D MPR and measuring the same on a 3D physical object. We found the same values under both the measurements which suffices that the segmentation is proper. This testing was done by an expert radiologist. We are working on other validation techniques. Figure 9 illustrates the visualization of the segmented volume in software, in STL object format before printing and a printed 3D model.

Table 2 shows the measurements of the tumor and anatomy in different directions on both software segmented object (X_i_) and on the 3D printed object (Y_i_). These measurements are w.r.t to one subject. Paired t-test was applied to see the differences in measurement. In group1, Mean=6.98, SD=2.4632 and in group 2, Mean=6.976, SD=2.4513. The mean of group1-group2=0.0040. The intermediate values used in the calculations were, t=0.3609, df=19 and standard error of difference=0.011. With this we got the two-tailed p=0.7222. Hence the measurements were same and the difference is considered to be not statistically significant. This proves that the segmentation algorithm results are correct.

It is possible to extend the same or refined segmentation technique with other anatomical tumor. We need more cases of occluded structures to test our methodology. It is possible to do random cuts in 3D visualization of the anatomies in computer software in any direction using clipping planes and the same directional information can be applied to do the similar cuts on the 3D printed object. This will help to study any complicated cases.

As we were more oriented towards realizing our idea of tumor quantification, we did not attempt for the parallel processing on GPU, now with this proof of concept we are planning to implementing with proper software design using object oriented concepts and coding using industry standard coding guidelines.

As we wanted to prove our idea of tumour validation, the research was started with the basic segmentation algorithms which are known already. And, since this is work in progress, and at the moment our framework is ready starting from reading the DICOM images, constructing the 3D volume, segmenting the VOI and printing the segmented object using STL format. Our further work will include exploring the best segmentation algorithms which would increase the accuracy of the tumour validation in both CT and MRI modalities.

Due to the cost constraint, only five segmented models were 3D printed in this study and the validation was performed and the results were convincing. With more dataset with different cases of brain tumour, the robustness of this method can be empirically tested.

## Discussion

In this paper, user-guided brain tumour segmentation for medical imaging datasets was carried out using the MITK framework. Connected threshold and Otsu’s algorithm was used to segment the tumour from the ROI. The connected threshold algorithm could identify the pixels that were similar using thresholds. The drawback of this method is that it ignored the spatial characteristics, and sometimes the tumour tissues were also ignored. Threshold-based segmentation is also limited to enhancing tumour areas. Otsu segmentation used histogram bins to classify the pixel into clusters based on their intensity values. If several clusters were used, the segmentation would have become more accurate, but this method is computationally expensive and took more time than the connected threshold method. The 3D printed model measurements and ground truth measurements are compared. 3D printing the model will also allow pre-operating on tumour will lead to a reduction in surgical time, decreased time under anaesthesia, and a dose of radiation. The computation times discussed in the time measured on CPU. We could not implement it on GPU. We have plans to redesign the complete work and plan to implement on GPU. Future work includes the addition of newer algorithms that use faster and accurate segmentation techniques and also a GPU implementation to speed up the computational process. The novelty of the proposed technique is, there is no other way to prove the accuracy of the segmentation results apart from the software means. Through this printing approach, we can produce the physical object from the virtual object and directly can be compared. This is one of the best approaches to prove the accuracy of the segmentation.

## Data Availability

The datasets analysed during the current study are available in the National Cancer Institute repository, https://public.cancerimagingarchive.net/ncia/login.jsf (http://doi.org/10.7937/K9/TCIA.2015.NWTESAY1).
